# Useful Biomarkers for Preeclampsia: Evaluating the Diagnostic Potential of FIB-4 and FIB-5 Indices

**DOI:** 10.3390/diagnostics15060693

**Published:** 2025-03-11

**Authors:** Mehmet Albayrak, Hilmi Furkan Arslan

**Affiliations:** 1 Department of Perinatology, Giresun Obstetrics and Pediatrics Training and Research Hospital, 28100 Giresun, Türkiye; 2 Department of Clinical Biochemistry, Giresun Obstetrics and Pediatrics Training and Research Hospital, 28100 Giresun, Türkiye

**Keywords:** preeclampsia, FIB-4 index, FIB-5 index, liver dysfunction, early detection

## Abstract

**Background/Objectives:** Preeclampsia is a systemic condition that can result in liver impairment and potentially lead to negative outcomes for both the mother and baby. Various indices have been created to facilitate the early detection of liver issues. Among these, the Fibrosis-4 index (FIB-4) and Fibrosis-5 index (FIB-5) have been utilized for several years to forecast liver dysfunction. In our research, we aimed to apply these indices to patients with preeclampsia for the first time. **Methods**: This retrospective study, conducted at Giresun University from 2017 to 2024, included 207 patients with preeclampsia and 205 healthy controls. Data on maternal age, BMI, obstetric history, delivery method, gestational age, birth weight, and Apgar scores were extracted from medical records for analysis and biochemical parameters. Data were analyzed statistically. **Results**: The study found that FIB-4 index values were significantly higher and FIB-5 index values were lower in preeclampsia patients compared to the controls. FIB-4 demonstrated a better diagnostic performance with higher sensitivity and specificity. Although the difference between the two indices was not statistically significant, both were independently predictive of preeclampsia. The correlation coefficient showed that FIB-4 was positively correlated with spot urine protein/creatinine ratio (SPCR) and aspartate aminotransferase (AST), while FIB-5 was negatively correlated with these parameters and with alkaline phosphatase. **Conclusions**: This study found that FIB-4 and FIB-5 are useful for predicting preeclampsia, with FIB-4 showing superior diagnostic performance. These findings highlight their potential in the early detection and management of preeclampsia. Further research is needed for clinical validation.

## 1. Introduction

Preeclampsia (PE) is a serious disorder of pregnancy, primarily believed to stem from abnormal placentation. It is initiated by reduced placental perfusion, which leads to systemic maternal syndromes. The condition typically manifests in the latter half of pregnancy as a systemic disorder characterized by hypertension, liver malfunction, and metabolic dysregulation. PE has long been associated with metabolic disturbances in the ischemic placenta. In early pregnancy, successful implantation and placentation depend on adequate trophoblast invasion and proper remodeling of uterine spiral arteries. However, accumulating evidence suggests that insufficient energy supply disrupts these processes, leading to abnormal implantation and superficial spiral artery remodeling. This, in turn, further disturbs the placental and fetal metabolome, creating a cycle of metabolic dysfunction. Maternal metabolism also plays a critical role, as it can exacerbate these events, while the placenta adapts its growth and fetal metabolism at the expense of the mother’s metabolic profile. Consequently, PE is closely linked to metabolic disorders such as dyslipidemia, hyperuricemia, hyperglycemia, and insulin resistance. These complications significantly contribute to maternal and fetal morbidity and mortality, underscoring the importance of early detection and intervention to prevent adverse outcomes [1,2,3,4].

Preeclampsia is the leading cause of liver dysfunction in approximately 3% of pregnancies due to the accumulation of microvesicular fat and decreased hepatic blood flow, which may lead to ischemia and periportal bleeding. Generally, levels of alanine aminotransferase (ALT) and aspartate aminotransferase (AST) stay within normal ranges; however, when these levels increase and are accompanied by abdominal discomfort, it strongly suggests a serious progression of the condition [5].

Various fibrosis indices have been developed to predict liver damage. The Fibrosis-4 (FIB-4) and Fibrosis-5 (FIB-5) indices are calculated using serum parameters commonly utilized to evaluate liver function [6,7]. As a result, they can be easily determined from routine blood tests conducted in clinical settings. The calculation of the FIB-4 takes into account the patient’s age, platelet count, and the levels of AST and ALT. The AST to Platelet Ratio Index (APRI) has been extensively studied concerning liver fibrosis and various systemic diseases [8]. In contrast to conventional biomarkers, FIB-5 index has been introduced more recently as a novel marker for evaluating liver dysfunction. The calculation is based on the values of albumin, alkaline phosphatase (ALP), the AST/ALT ratio, and platelet count, indicating its potential for forecasting outcomes in patients with vascular and inflammatory conditions components [9]. The FIB-4 and FIB-5 are both cost effective and readily accessible, making them valuable tools for primary-care physicians in the evaluation of liver fibrosis during routine clinical practice [7].

Recent research has explored associations between acute fatty liver disease of pregnancy (AFLP), intrahepatic cholestasis of pregnancy (ICP), and preeclampsia [10,11,12]. To the best of our knowledge, only one study has investigated the relationship between the FIB-4 index and preeclampsia [13]. However, no prior studies have examined the association between the FIB-5 index and preeclampsia. Consequently, our study represents the first investigation of this relationship in the literature. This study aims to investigate how the FIB-5 and FIB-4 indices relate to maternal and fetal outcomes.

## 2. Materials and Methods

### 2.1. Ethics Approval

This study was conducted at the Giresun University Obstetrics and Gynecology and Pediatric Diseases Training and Research Hospital from 2017 to 2024. The Ethics Committee of Giresun University approved the study protocol, which complied with the requirements of the Declaration of Helsinki (approval number: 7, date: 8 May 2024).

### 2.2. Study Design, Patient Selection, and Diagnosis

This retrospective study included 205 healthy pregnant women without complications and 207 pregnant women with preeclampsia. After the 20th week of pregnancy, hypertension, proteinuria, and hyperuricemia were used to diagnose preeclampsia. A blood pressure reading of at least 140 mmHg in the systolic phase and 90 mmHg in the diastolic phase, measured at least four hours apart, was considered to represent hypertension. A dipstick test of ≥2+, a 24 h urine protein level of ≥0.3 g/day, or a spot urine protein/creatinine ratio > 30 mg/mmol were used to diagnose proteinuria [2].

The exclusion criteria were pregnancies under 20 weeks, multiple gestations, women in active labor, or those who could not be monitored. Additionally, women with adverse maternal outcomes before the collection of biochemical data and eligibility criteria were excluded from the study.

Information regarding age, body mass index (BMI), history of pregnancies, live births, and complications in earlier pregnancies was collected from medical records. Other documented variables included the diagnosis, gestational age at the time of delivery, method of delivery, birth weight, and Apgar scores.

FIB-4 is computed using this formula [14]:

age (years) × AST (IU/L)/[platelet count (×10^9^/L) × ALT (IU/L)^1/2^]

FIB-5 is computed using this formula:

[albumin (g/L) × 0.3 + platelet count (10^9^/L) × 0.05] − [ALP (IU/L) × 0.014 + AST to ALT ratio × 6 + 14]

### 2.3. Statistical Analyses

MedCalc version 20.115 (MedCalc Software Ltd., Ostend, Belgium) and SPSS version 26.0 (IBM Corp., Armonk, NY, USA) were used for the statistical analyses. We used the Shapiro–Wilk and Kolmogorov–Smirnov tests to check if the continuous variables followed a normal distribution. The results are presented as the mean ± standard deviation for variables with a normal distribution (parametric data) and as the median (25th–75th percentile) for variables with a non-normal distribution (non-parametric data). Categorical data are expressed as numbers (percentages). A chi-squared test was employed to analyze categorical data. For normally distributed data, a Student’s *t*-test was used, while a Mann–Whitney U test was applied for non-normally distributed data to compare cases of preeclampsia with the control-group groups.

We assessed the diagnostic accuracy of the tests through receiver operating characteristic (ROC) analysis, utilizing the Youden index to establish the optimal cut-off value. To assess and compare the diagnostic efficacy of the tests, pairwise comparisons of the ROC curves were performed by comparing the area under the curve (AUC) values. The relationships between FIB-4, FIB-5, and other laboratory data were investigated using Spearman’s correlation test. A binary logistic regression was performed to evaluate the independent risk factors for preeclampsia associated with the FIB-4 and FIB-5 scores. The threshold for statistical significance was established at *p* < 0.05.

## 3. Results

This research involved 207 pregnancies affected by preeclampsia and 205 healthy control pregnancies. This study included 207 pregnancies affected by preeclampsia and a control group of 205 healthy pregnancies. A comparison was made of the demographic, clinical, and perinatal characteristics between the two groups. There were no significant differences in maternal age, gravidity, or parity between the preeclampsia and control groups (*p* > 0.05). Nevertheless, women with preeclampsia had a significantly earlier gestational age at delivery and lower measurements in terms of birth weight, birth length, and Apgar scores at both one and five minutes compared to the control group. Additionally, the rate of cesarean deliveries was significantly higher in the preeclampsia group (*p* < 0.05) (Table 1).

Table 2 presents a comparison of the laboratory findings between the preeclampsia and control groups. The ALT, AST, ALP, and FIB-4 index were significantly higher in the preeclampsia group, whereas the FIB-5 score was significantly lower than that in the control group (*p* < 0.05) (Table 2).

**Figure 1:**
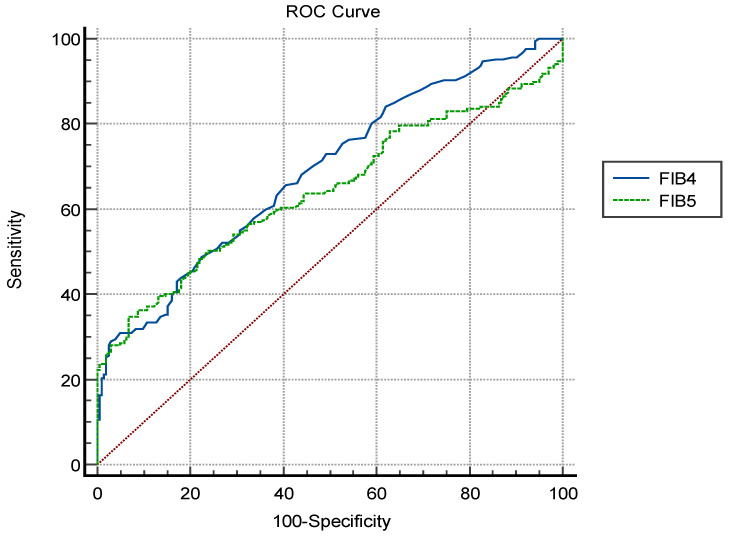
The ROC curves of FIB-4 and FIB-5 for predicting preeclampsia.

A pairwise comparison of the ROC curves was conducted to compare the diagnostic performances of these tests, revealing that, although the AUC value for FIB-5 was lower than that for FIB-4 (*p* = 0.001) (Table 3, Figure 1).

In the preeclampsia group, the Spearman correlation coefficient demonstrated a positive correlation between the spot urine protein/creatinine ratio (SPCR) (r = 0.695; *p* = 0.001) and AST levels (r = 0.536; *p* = 0.001). Conversely, FIB-4 was negatively correlated with FIB-5 (r = −0.733; *p* = 0.001) and ALT (r = −0.196; *p* = 0.001) (Table 4).

In the univariate regression analysis, both FIB-4 and FIB-5 scores were significantly associated with preeclampsia. Furthermore, after adjusting for maternal age, gestational age at delivery, systolic blood pressure, and diastolic blood pressure, the association between FIB-4 and FIB-5 scores and preeclampsia remained significant in the multivariate regression analysis (Table 5).

## 4. Discussion

This research aimed to evaluate the diagnostic value of the FIB-4 and FIB-5 indices to enhance the understanding and management of preeclampsia. The findings indicate that these indices, particularly FIB-4, are effective in distinguishing cases of preeclampsia from normal pregnancies. Among these metrics, FIB-4 demonstrated greater diagnostic accuracy in predicting preeclampsia. Although FIB-5 also offers diagnostic potential, its ability to discriminate appears to be less effective compared to FIB-4. These results underscore the promise of FIB-4 as a significant biomarker for evaluating the risk of preeclampsia and enhancing patient care.

Preeclampsia is a complicated condition of pregnancy that affects several organ systems and poses serious dangers to the health of both the mother and the fetus. According to previous studies, it is one of the main factors contributing to maternal morbidity and mortality. Owing to the heterogeneity of its clinical presentation, it is still difficult to predict which women may develop severe preeclampsia, even with breakthroughs in care [15].

Although the precise cause of preeclampsia remains unclear, aberrant placentation is believed to result in endothelial dysfunction and placental hypoperfusion. As pregnancy progresses, faulty trophoblast invasion impairs arterial placental perfusion, which causes endothelial damage, systemic inflammation, and the release of vasoactive substances such as prostaglandins, endothelin, and nitric oxide. This results in hypertension, platelet aggregation, and vasoconstriction. Microangiopathic hemolytic anemia may arise from fibrin deposition in small blood vessels in response to endothelial damage. Preeclampsia-related liver involvement most likely results from fibrin accumulation in the hepatic sinusoids, which can cause hepatic ischemia, parenchymal bleeding, subcapsular hematomas, or hepatic rupture in extreme circumstances [1,2,3].

The liver undergoes physiological alterations throughout normal pregnancy to meet the growing fetus’s increasing metabolic needs. Liver function is affected by elevated levels of progesterone and estrogen, which increase the levels of alpha-fetoprotein (AFP) and ALP. Hemodilution usually results in a decrease in certain liver enzymes, including γ-glutamyl transferase (GGT), ALT, and AST. In contrast, the kidneys, liver, brain, and placenta experience ischemia because of vascular abnormalities linked to preeclampsia [5]. Histological results reported by Minamaki et al. [16] showed that the livers of preeclamptic women had fibrinogen accumulation, periportal hemorrhage, and microvesicular fat. This is because the subendothelial collagen is exposed to endothelial cell damage. Although hemolysis is the primary symptom of postpartum hemorrhage, endothelial dysfunction caused by uteroplacental ischemia is the cause of the clinical manifestations of hemorrhage hemolysis, including coagulopathy, proteinuria, edema, and hypertension due to the release of inflammatory mediators [17,18,19]. The observed damage to the liver may be explained by an imbalance between proangiogenic and antiangiogenic agents [20]. When compared to normotensive controls, preeclamptic patients had higher levels of fibrosis in FibroScan research [21], which has also been noted in liver biopsies conducted due to changes in lipid metabolism. In hepatic subcellular organelles, microvesicular steatosis results in oxidative stress and mitochondrial dysfunction [22]. Preeclamptic patients have demonstrated serious consequences that affect 4–12% of women with preeclampsia, “Hemolysis, Elevated Liver enzymes, Low Platelet count” (HELLP) syndrome, and liver malfunction. Such situations result in noticeably higher liver enzyme levels and decreased platelet counts, thereby increasing the possibility of maternal mortality from bleeding. Acute fatty liver of pregnancy (AFLP), an uncommon but deadly condition that can cause acute liver failure and maternal death, is a major liver-related concern. Because AFLP is more common in areas where obesity is very prevalent, it is crucial to monitor liver function in at-risk women.

The predictive potential of liver function markers for preeclampsia has been investigated in several recent studies. Preeclampsia in the early stages of pregnancy may be predicted by GGT, ALP, and the AST/ALT ratio, as reported by Liu et al. [23]. The onset of preeclampsia was positively correlated with elevated GGT and ALP levels but negatively correlated with the AST/ALT ratio. It has also been demonstrated that the hepatic steatosis index, which considers BMI and liver enzyme levels, is predictive of preeclampsia [24].

APRI and FIB-4 are two examples of liver fibrosis scores that have been used to examine liver fibrosis in chronic liver diseases [7,25]; however, more recent studies have explored their wider use, such as their potential to predict systemic inflammation and endothelial dysfunction in diseases such as preeclampsia. It has been extensively utilized as a non-invasive marker for liver fibrosis in non-alcoholic fatty liver disease (NAFLD) [14,26]. Additionally, it has demonstrated potential in forecasting hospital mortality among patients with heart failure and acute coronary syndrome [19]. Likewise, the FIB-5 index can anticipate results in cardiovascular conditions.

Ozkan et al. [27] found that APRI scores were significantly elevated in the preeclampsia group before 20 weeks of gestation, supporting its role as an early indicator. Similarly, Zhaoqi et al. [28] observed that individuals in the HELLP group exhibited significantly higher levels of transaminases and platelet counts compared to those with isolated prenatal hypertension, based on a study involving 465 patients diagnosed with preeclampsia complicated by HELLP syndrome. The APRI1 score was lower in the superimposed preeclampsia group compared to both the control group and the hypertension group, while APRI2 levels were found to be higher in the superimposed preeclampsia group in comparison to the control group. İpek et al. [29] analyzed APRI scores in 167 patients suffering from chronic hypertension, evaluating its utility in predicting superimposed preeclampsia.

Another retrospective analysis by Özer et al. [13] found that women with mild preeclampsia had significantly higher FIB-4 scores compared to the healthy control group, which had lower values. Furthermore, FIB-4 scores were lower in women with mild preeclampsia than in women with severe preeclampsia, indicating a correlation between preeclampsia severity and increasing FIB-4 scores. These findings and the findings of the studies by Kolhe et al. [30] and Kumari et al. [31] are consistent with our results.

In contrast to our findings, multiple studies have indicated that the FIB-5 index serves as a more effective predictor for heart failure, chronic hepatitis B and C, and non-alcoholic fatty liver disease [14,32,33]. Additionally, FIB-5 has been demonstrated to forecast outcomes in conditions like portal hypertension and NAFLD [3,26]. The noted differences may stem from the distinct pathophysiological mechanisms of preeclampsia as compared to other liver diseases.

### Study Limitations

This research presents various limitations. First, although the sample size was moderate, it may not be sufficient to generalize the findings to a wider population of preeclampsia patients. Moreover, because the study was conducted at a single center, there is a possibility of selection bias, which could limit the external validity and generalizability of the results. The retrospective nature of the study also poses inherent limitations, such as potential confounding variables that were not controlled for or missing data that could affect the outcomes. Finally, liver fibrosis indices were only assessed at a specific point in time, and longitudinal data are necessary to better understand their role in the progression of preeclampsia. Additional research involving larger and more diverse populations and prospective study designs is necessary to confirm and validate these findings.

## 5. Conclusions

This study demonstrated that FIB-4 levels were significantly elevated and FIB-5 levels were significantly decreased in women with preeclampsia, suggesting their potential role as diagnostic markers in this condition. Although the diagnostic performance of FIB-4 was slightly superior to FIB-5 in terms of sensitivity and specificity, the difference was not statistically significant. These indices, a cost-effective and accessible tool traditionally used to grade liver fibrosis, may serve as a valuable diagnostic adjunct for preeclampsia, especially given their ease of application in routine clinical practice. However, our study should be considered a pilot study, as it was a case–control study, and larger-scale, prospective studies are needed to confirm these findings and investigate their broader impact in improving maternal and perinatal outcomes

## Figures and Tables

**Table 1:** Demographic, clinic, and perinatal characteristics of the preeclampsia patients and controls.

	Preeclampsia (*n* = 207)	Control (*n* = 205)	*p*-Value
Maternal age	31.2 ± 6.89	29.5 ± 5.13	0.067 ^a^
Gravidity	3.1 ± 1.9	2.9 ± 1.3	0.18 ^a^
Parity	1.8 ± 0.9	1.5 ± 1.1	0.36 ^a^
Systolic blood pressure (mmHg)	156.5 ± 9.2	110.6 ± 7.3	**<0.001** ^a^
Diastolic blood pressure (mmHg)	96.8 ± 7.5	63.5 ± 6.8	**<0.001** ^a^
Cesarean delivery	182 (87%)	56 (27%)	**<0.001** ^b^
Vaginal delivery	26 (13%)	149 (73%)	**<0.001** ^b^
Gestational age at delivery (weeks)	35.1 ± 1.7	38.2 ± 0.6	**0.034** ^a^
Birth weight (grams)	2511 ± 593	3176 ± 684	**<0.001** ^a^
Birth length (cm)	44.16 ± 3.98	50.21 ± 4.79	**<0.001** ^a^
First-minute Apgar score	7.6 ± 1.2	8.1 ± 0.8	**0.025** ^a^
Fifth-minute Apgar score	8.7 ± 0.9	9.2 ± 1.1	**0.036** ^a^

^a^ Student’s *t*-test was used; data are presented as the mean ± SD. ^b^ Chi-squared test was used; data are presented as the ratio (percentage). Values in bold are statistically significant (*p* < 0.05).

**Table 2:** Laboratory findings of the preeclampsia patients and controls.

	Preeclampsia (*n* = 207)	Control (*n* = 205)	*p*-Value
Hemoglobin (g/dL)	11.50 (10.50–12.60)	11.80 (11.01–12.75)	**0.024** ^a^
Platelet (×10^3^/mm^3^)	220 (181.5–286.5)	225 (194–269)	0.643 ^a^
Alanine aminotransferase (U/L)	19.2 (8.4–29.7)	10.9 (6.3–15.7)	**<0.001** ^a^
Aspartate aminotransferase (U/L)	21.7 (14.2–33.5)	16.1 (13.8–19.4)	**<0.001** ^a^
Alkaline phosphatase (U/L)	138.6 (103.5–184.5)	116.3 (91.1–137.8)	**0.037** ^a^
Serum albumin (g/L)	35.4 (32.3–38.7)	37.3 (36.2–40.5)	0.164 ^a^
Serum creatinine (mg/dL)	0.58 ± 0.19	0.53 ± 0.12	0.058 ^b^
Spot urine protein/creatinine ratio	0.72 ± 0.25	0.14 ± 0.07	**<0.001** ^b^
FIB-4 score	0.86 ± 0.39	0.63 ± 0.24	**<0.001** ^b^
FIB-5 score	−3.34 (−7.33–1.43)	−1.92 (−4.13–0.79)	**<0.006** ^a^

^a^ Mann–Whitney U test was used; data are presented as the median and interquartile range (25–75%). ^b^ Student’s *t*-test was used; data are presented as the mean ± SD. Values in bold are statistically significant (*p* < 0.05). FIB-4: Fibrosis-4; FIB-5: Fibrosis-5.

**Table 3:** Receiver operating characteristic analysis of FIB-4 and FIB-5 scores for predicting preeclampsia.

	AUC *	95% Confidence Interval		Cut-Off Value	Sensitivity	Specificity	*p* Value
		Lower	Upper				
FIB-4	0.689	(0.642–0.733)		>0.78	58.7	77.5	<0.001
FIB-5	0.643	(0.589–0.697)		<−3.13	54.1	69.2	<0.001
Comparison of AUCs			AUC				
			Difference		95% Confidence interval		
FIB-4 and FIB-5			0.046		(0.005–0.098)		0.081

* AUC: area under the curve.

**Table 4:** Spearman correlation coefficient between FIB-4, FIB-5, and other laboratory parameters.

		SPCR	ALT	AST	ALP	FIB-5	FIB-4
FIB-4	r	0.695	−0.196	0.536	0.027	−0.733	
	*p*	**<0.001**	**0.042**	**<0.001**	0.588	**<0.001**	(–)
FIB-5	r	−0.542	0.274	−0.246	−0.190		−0.733
	*p*	**<0.001**	**<0.001**	**<0.001**	**<0.001**	(–)	**<0.001**

r: Spearman correlation coefficient. SPCR: spot urine protein/creatinine ratio. ALT: alanine aminotransferase. AST: aspartate aminotransferase. ALP: alkaline phosphatase. Values in bold are statistically significant (*p* < 0.05).

**Table 5:** Binary logistic regression analysis results for preeclampsia.

Univariate Regression (Unadjusted)				Multivariate Regression (Adjusted)			
Parameter	OR	%95 CI	*p* Value	Parameter	OR ^a^	%95 CI	*p* Value
FIB-4	2.178	(1.436–3.479)	<0.001	FIB-4	1.936	(1.367–3.015)	**<0.001**
FIB-5	0.917	(0.825–0.984)	<0.001	FIB-5	0.724	(0.665–0.813)	**<0.001**

^a^ Odds ratio adjusted for maternal age, gestational age at delivery, systolic blood pressure, and diastolic blood pressure. OR: odds ratio. CI: confidence interval. Values in bold are statistically significant (*p* < 0.05).

## Data Availability

The datasets used and/or analyzed in this study are accessible from the corresponding author upon reasonable request.
